# Morphology and Mechanical Properties of Polyimide Films: The Effects of UV Irradiation on Microscale Surface

**DOI:** 10.3390/ma10111329

**Published:** 2017-11-20

**Authors:** Changzi Qu, Junsong Hu, Xing Liu, Zheng Li, Yanhuai Ding

**Affiliations:** 1 Institute of Rheological Mechanics, Xiangtan University, Xiangtan 411105, China; quchangzi83@xtu.edu.cn (C.Q.); junsonghu@hotmail.com (J.H.); 13873219217@163.com (X.L.); 2 Key Laboratory of Intelligent Computing & Information Processing of Ministry of Education, Xiangtan University, Xiangtan 411105, China

**Keywords:** polyimide, UV irradiation, roughness, mechanical properties, FEM, damage evaluation, AFM

## Abstract

As an attractive dielectric material, polyimide has been widely used in the field of electronics, aerospace, and automobiles due to its useful mechanical properties and good chemical resistance. UV irradiation was considered to be the main factor related to the damage and failure of polyimide. Here the effects of UV irradiation on the surface morphology and microscale mechanical properties of polyimide films are characterized by atomic force microscopy (AFM). The surface roughness of the UV-irradiated samples developed and the mechanical properties degraded with the radiation dose increased. For comparison, uniaxial tensile test was performed to obtain the macroscale Young’s modulus of polyimide film. The UV-irradiated damaging depth was simulated with finite element method (FEM).

## 1. Introduction

Polyimide (also known as PI or Kapton) plays an important role in aerospace and microelectronics due to its attractive dielectric properties, mechanical strength, excellent thermal stability, and chemical properties [1,2,3,4,5]. PI film with excellent physical, electrical, and mechanical properties over a wide temperature range (from −269 °C to 400 °C) has opened up new design and application areas to plastic films [6]. PI film is ideal for insulating circuit boards, high-temperature powder coating, and transformer manufacturing due to the desirable properties of high temperature resistance and good dimensional stability [7,8]. For example, PI film was used as a low-cost, flexible interface between the flip-chip bonded sensors at one end and wires to external electronics at the other end [9]. A microwave/microfluidic sensor was fabricated on a flexible PI substrate for complex permittivity characterization of liquids [10]. A wide range of nanostructures and nanosensors have been grown successfully on the PI film [11,12,13]. It has been recognized that PI film can be employed as a flexible substrate for modern electronic devices. 

As a ubiquitous environmental factor, UV irradiation was considered a natural enemy of almost all polymers exposed to sunlight. PI has been always subject to various adverse factors during its long-term service, including environmental corrosion, material aging, etc. Among them, UV-induced aging was thought to be one of the primary causes of failure. PI shows a broad UV absorption band in the UV region between 180 and 400 nm, allowing the effective absorption of all common excimer laser wavelengths [14]. The effects of the exposure of ultraviolet (UV) excimer light on the physicochemical surface properties of PI films have been widely investigated [14,15]. UV photons are energetic enough to dissociate molecular bonds, resulting in photo-oxidation reactions that scissor or crosslink the polymer chains. The contact angles of water on PI films decreased remarkably after exposure to 172 nm excimer light in ambient air, which was attributed to the increase in oxygen concentration at the film surface [16]. UV irradiation was also employed for surface modification of PI [17]. The mechanical property is a major index for the application of PI. However, the mechanical behavior and damage evolution of PI films exposed to UV light have rarely been reported [18]. Here we investigated the effects of UV irradiation on the surface morphology and microscale mechanical properties of PI films by atomic force microscopy. The UV-irradiated damaging depth was simulated by the finite element method (FEM). 

## 2. Experimental

### 2.1. Materials

**Figure 1:**
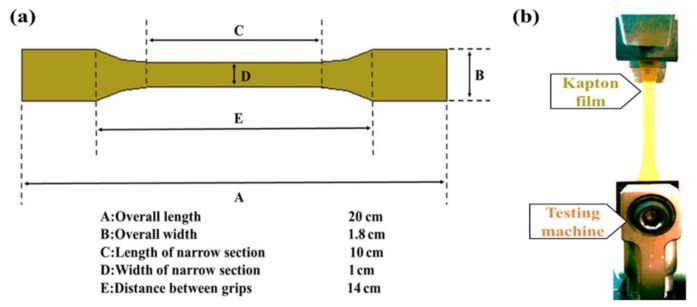
(**a**) Dimensional drawings of dumbbell-shaped specimen; (**b**) digital photo of uniaxial tensile tests.

PI films purchased from DuPont (Shanghai, China) in the form of individual sheets with a thickness of 0.05 mm were used in our work (for details see Table S1). Before being subjected to UV irradiation, the films were washed in ultrasound by using both acetone and isopropanol. Then the films were exposed to a high-pressure mercury lamp in the air at a distance of 15 cm. The UV intensity measured by a UV-meter was 125 μW/cm^2^. After UV irradiation, the samples were cleaned in alcohol for 5 min with ultrasound, then dried in a vacuum at 60 °C for the AFM characterization. At the same time, as shown in Figure 1, dumbbell-shaped specimens were subjected to an Instron 5940 universal testing machine (Shanghai, China) for tensile testing. The uniaxial tensile tests were conducted at a constant elongation rate of 0.05 mm/s. The morphological evolution and structure were investigated by atomic force microscopy (AFM, Veeco Multimode 8, Santa Barbara, CA, USA) and Fourier transform infrared (FTIR) spectroscopy (Perkin Elmer, Shanghai, China).

### 2.2. Methods

**Figure 2:**
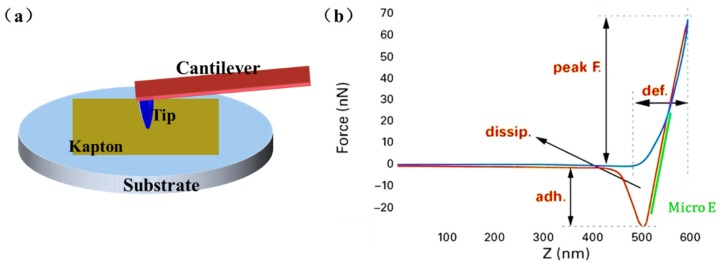
(**a**) Mechanical characterization of PI films by PFQNM technology; (**b**) typical force curve from PFQNM.

Peak-force QNM (PFQNM) mode, a newly developed AFM technology integrating the Tapping and QNM modes, can capture the topography of the sample and calculate the mechanistic properties at each point of the scan area [19,20,21]. The PFQNM mode has been widely used for the investigation of mechanical properties of materials at microscale to nanoscale, which can obtain the modulus directly without further manual calculation or simulation. Figure 2a represents the morphology and nanomechanical characterization of the UV-irradiated PI films examined by AFM with PFQNM mode. As illustrated in Figure 2b, the microscale elastic modulus *E* is obtained by fitting the classical Hertzian contact model to the force curves. The area between the blue approach curve and the red retraction curve reflects the energy dissipation. The distance that the tip was indented into the sample is reported as the deformation. A silicon probe tip (RTEST, Bruker, Karlsruhe, Germany) with spring constant of ~40 N/m was used. The contact radius of 20 nm was calibrated by the standard samples. The measurements of roughness, microscale elastic modulus, and Young’s modulus of the samples were taken six times and then averaged.

FEM numerical simulation was performed to forecast the damaging depth in PI films (for details see the Figure S1 and Table S2). The finite element models of UV-irradiated samples were divided into damaged and undamaged layers in the thickness direction. The materials of two layers were all considered as elastic linear isotropic solids to simulate the uniaxial tensile test in the elastic range. From the results of uniaxial tension tests and numerical simulation, the thickness of each layer was figured out.

## 3. Results and Discussion

**Figure 3:**
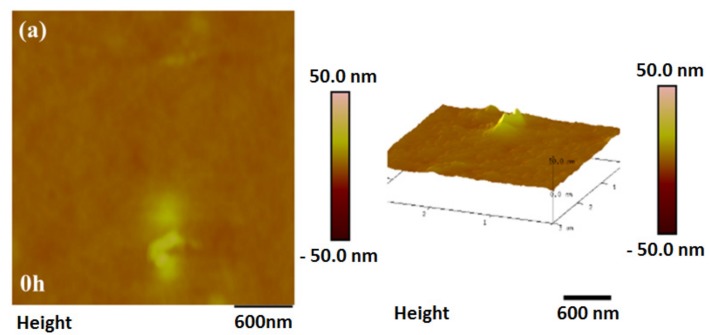

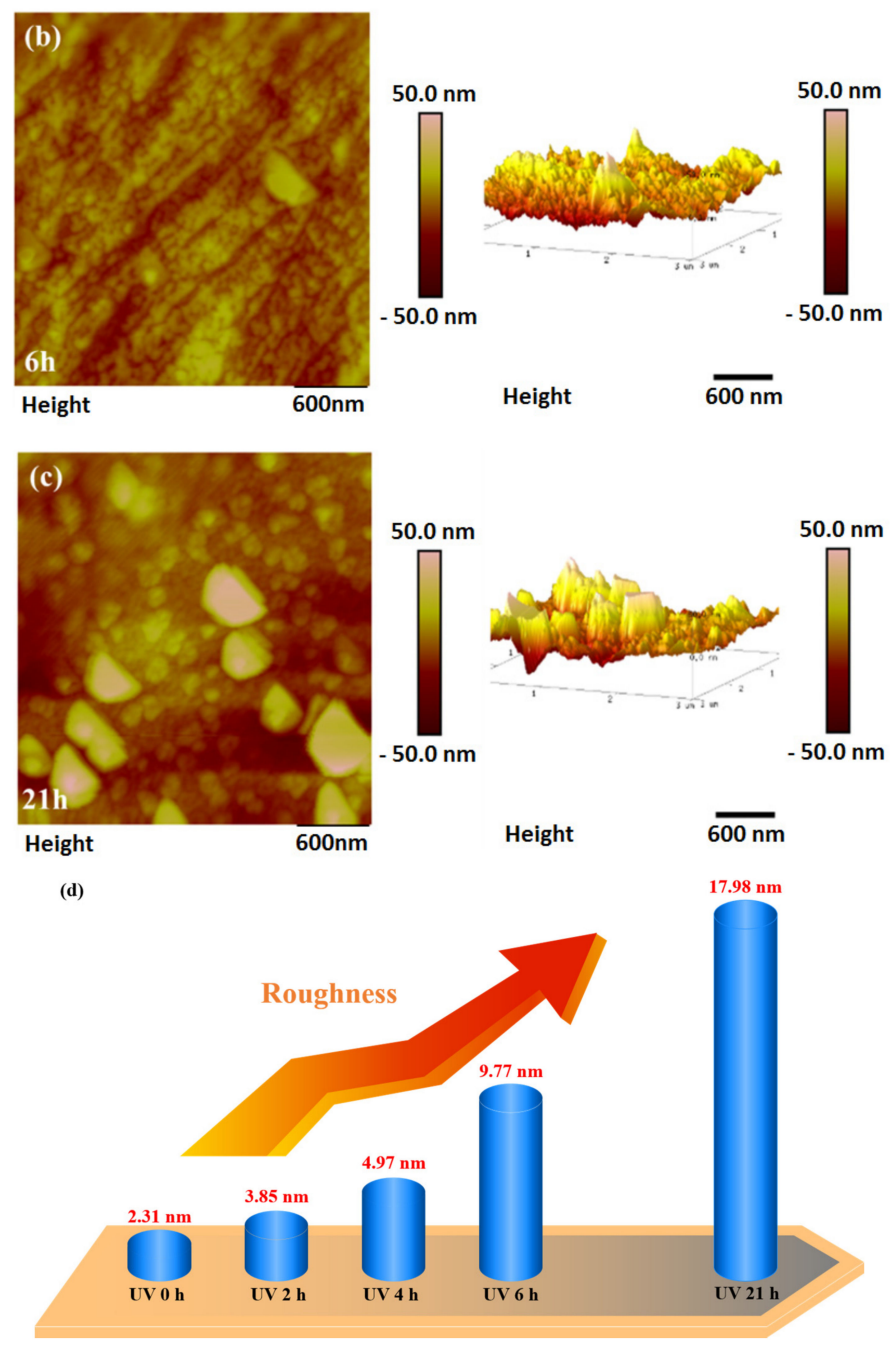
The surface morphology and roughness of PI films with different UV irradiation times. (**a**) irradiated for 0 h; (**b**) irradiated for 6 h; (**c**) irradiated for 21 h; (**d**) the roughness of PI films at different UV irradiation times.

The exposed surfaces of PI film were observed by AFM, as shown in Figure 3. Numerous fine bumps formed on the surface by UV irradiation. The size of the bumps increased with the increasing radiant dose. High-energy photons generated from UV light acted on the chemical valence bonds of polymers, which led to the cleavage and crosslinking of polymer chains [22]. After 21 h of irradiation, some particles were formed with a diameter of ~20 nm. A possible explanation could be the degradation of the PI to lower molecular weight species and carbon particles, which built a homogeneous layer on the surface of PI film. Lower-energy bonds like C–C, C–O, and C–N in PI films would break after absorbing a specific UV wavelength [23,24]. The surface structure was confirmed by FTIR as shown in the Figure S2. As shown in Figure 3d, the roughness increased remarkably with the radiant time, agreeing well with the morphological characterization. 

**Figure 4:**
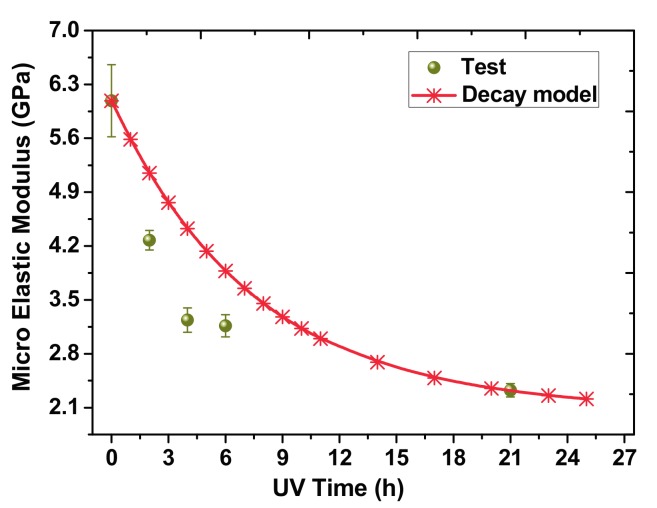
Microscale *E* of PI films at different UV irradiation times.

The changes of microscale elastic modulus *E* of the samples under UV irradiation are demonstrated in Figure 4. The UV-irradiated samples underwent a gradual decrease in their tensile properties as the UV radiant dose increased. In order to quantitatively characterize the variation of *E*, curve fitting procedures were used to analyze the tests results as follows:

(1) $$E_{t}=(1-D_{1})E_{0}$$

where $E_{t}$ is the microscale elastic modulus after *t* hours irradiation and $E_{0}$ is the initial elastic modulus of the surface. *D*_1_ is a time-dependent parameter related to the physical properties of materials ($D_{1}=66(1-e^{-\frac{t}{7.5}})\%$). As shown in Figure 4, the $E_{t}$ decreased dramatically with increasing irradiation time at first, and then stabilized at $E_{0}$. 

**Figure 5:**
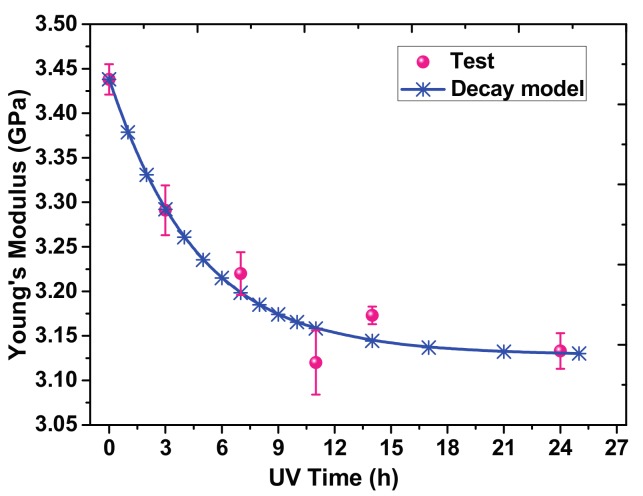
The Young’s modulus of PI films at different UV irradiation times.

Figure 5 presents the Young’s modulus of the PI films at different irradiation times. The fitting function of Young’s modulus measured by uniaxial tensile tests is similar to the microscale elastic modulus:

(2) $$E_{t}^{'}=(1-D_{2})E_{0}^{'},$$

where $E_{t}^{'}$ is the Young’s modulus after *t* hours of irradiation, and $E_{0}^{'}$ is the initial Young’s modulus of the PI film. *D*_2_ is a time-dependent parameter related to the physical properties of materials ($D_{2}=9(1-e^{-\frac{t}{4.7}})\%$). After prolonged irradiation, the Young’s modulus stabilized around 3.13 GPa. Obviously, the effect of UV irradiation on the microscale elastic modulus is more remarkable than the Young’s modulus. The irradiated specimens are divided into two parts: the damaged and the undamaged layer in the thickness direction. The damaged zone can be considered to continuously expand inward from the irradiated surface, eventually reaching the whole specimen. The Young’s modulus of the damaged layer eventually reached 3.13 GPa and the Young’s modulus of the undamaged layer stayed at 3.45 GPa. The rapid decrease of elastic modulus on the surface could be ascribed to the flowability of polymer materials. The amplitude of the thermal vibrations of molecules and the thermal expansion coefficients at the surface are much larger than in the bulk, which increased the fluidity of the surface when subjected to external loading [25]. 

**Figure 6:**
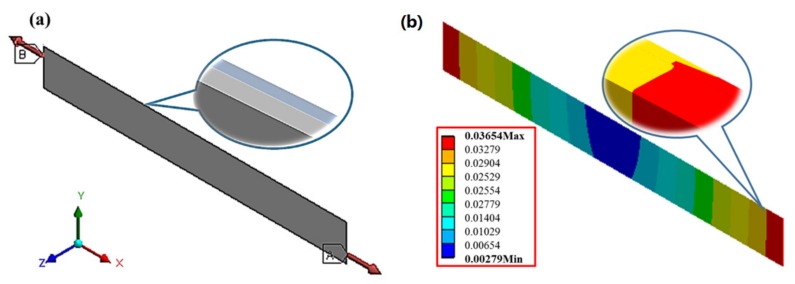
(**a**) The finite element model of uniaxial tension established in ANSYS 15.0; (**b**) the elongation diagram from FE numerical simulation.

Figure 6 shows the finite element model of uniaxial tension established in a commercial software package (ANSYS 15.0, Company, Canonsburg, PA, USA) [26,27]. The details of the structural model and simulation are given in the Figure S1. The upper layer is the damaged layer and the lower is the undamaged one. It should be noted that the thickness of each layer is unknown. Thus, it is essential to perform an exploratory calculation in the initial stage of numerical simulation, changing the thickness of each layer constantly until the numerical simulation results are consistent with the uniaxial tensile tests. Then the thickness of the damaged layer at different UV irradiation times can be calculated.

**Figure 7:**
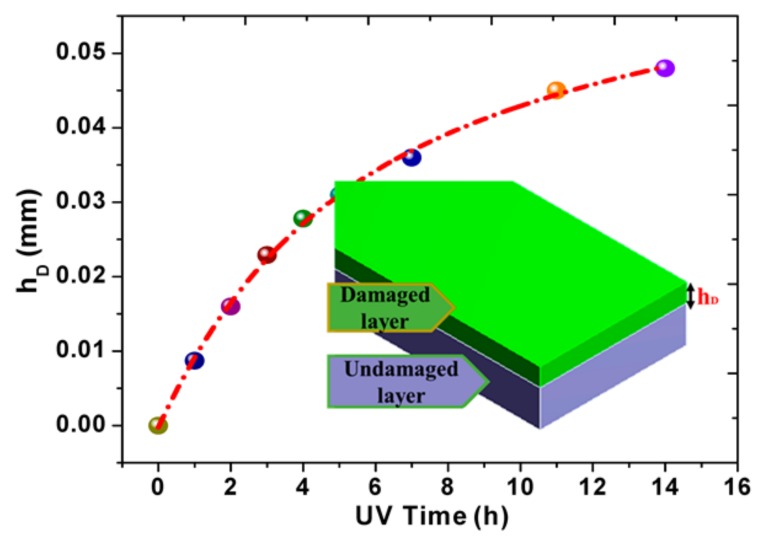

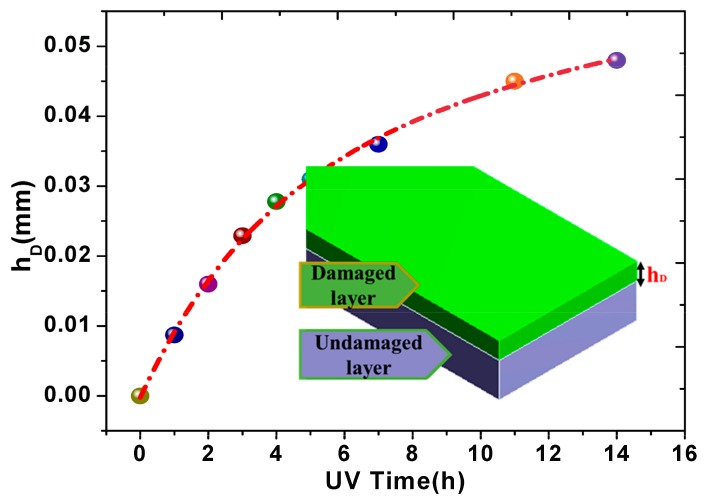
The damaged depth in PI films at different UV irradiation times.

The variation of damaged depth with UV irradiation time is depicted in Figure 7. The following empirical function is satisfied:

(3) $$h_{D}=\left[0.07-(0.03e^{-\frac{t}{3.54}}+0.04e^{-\frac{t}{23.42}})\right],$$

where *t* is the UV irradiation time and $h_{D}$ is the thickness of UV irradiation damaged layer. The damaged depth increased faster in the early stage and then the increasing extent is gradually reduced. After 14 h, the damage zone has spread to the whole film. That is to say, the Young’s modulus of UV-irradiated specimens will tend to be stable, which is perfectly consistent with the uniaxial tension tests above. It means the damaging depth at different radiation times could be established based on the physical parameters related to the polymers. 

## 4. Conclusions

In the present work, the surface roughness of the UV-irradiated samples increased and the mechanical properties degraded with the increasing radiation dose. The damage depth at different radiation times could be established based on the physical parameters related to the polymers. This provides a new way to evaluate the impact of UV irradiations on polymer materials.

## Supplementary Materials

The following are available online at www.mdpi.com/1996-1944/10/11/1329/s1, Figure S1: The model in finite element method, Figure S2: FTIR spectra of PI film before and after UV irradiation, Table S1: The main properties and characteristics of PI, Table S2: The Material Parameters for simulation.
