# Supplementary material for: Morphology and Mechanical Properties of Polyimide Films: The Effects of UV Irradiation on Microscale Surface
Source: Materials (Basel). 2017 Nov 20;10(11):1329. doi: 10.3390/ma10111329 (PMC5706276; doi:10.3390/ma10111329)
Supplement: Supplementary file 1 [file materials-10-01329-s001.pdf]

Supporting Information for 'Morphology and Mechanical Properties of Polyimide Films: the Effects of UV Irradiation on Microscale Surface'

Changzi Qu [1], Junsong Hu [1], Xing Liu [1], Zheng Li [1,*] and Yanhuai Ding [1,2,*]

[1] Institute of Rheological Mechanics, Xiangtan University, Xiangtan 411105, China; quchangzi83@xtu.edu.cn (C.Q.); junsonghu@hotmail.com (J.H.); 13873219217@163.com (X.L.)

[2] Key Laboratory of Intelligent Computing & Information Processing of Ministry of Education, Xiangtan University, Xiangtan 411105, China

* Correspondence: yhding@xtu.edu.cn (Z.L.); lizheng0121@hotmail.com (Y.D.)

The PI film used dose not contain any filler. The main properties and characteristics of PI film are listed in Table S1.

Table 1 The main properties and characteristics of PI

Properties	Units	Values
Thickness	mm	0.05
Density	g/cm^3	1.42±0.2
Elastic Modulus	GPa	3-4
Tensile strength	Mpa	>100
Temp resistance	^{o}F	-452-752
Coefficient of thermal expansion	1/ ^{o}F	2*10^{-5}-3*10^{-5}

A finite element method 3D model of multilayer material is built as shown in Fig.S1. The model is divided into damaged and undamaged layers in the thickness direction. The materials of two layers were all considered as elastic linear isotropic solids to simulate the uniaxial tension test of elastic range, their material parameters are presented in Table.S2. The specimen geometry is meshed using 8-node continuum elements, which are compatible for the analysis. It is noteworthy that, in order to ensure the accuracy of calculation, five layers of elements are used to model the through thickness of 0.05 mm as shown in the insert of Fig.S1. Additionally, the dimensions of the model are identical with narrow section of dumbbell shaped specimen prepared in uniaxial tension tests, that is 10mm high, 100mm long and the thickness is 0.05mm.

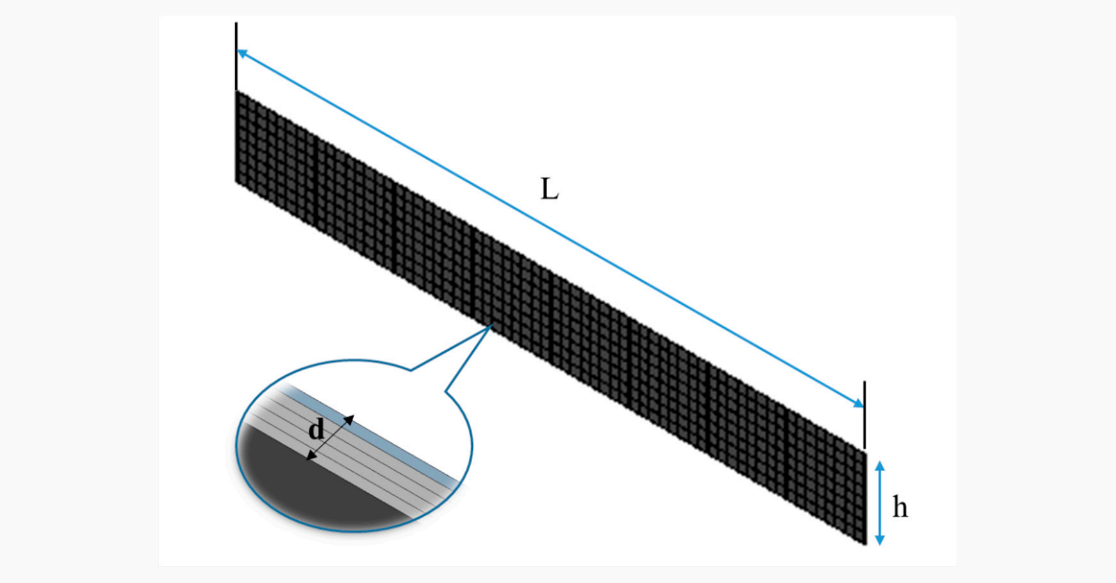

Fig.S1 The model in finite element method

Table.S2 The Material Parameters for simulation

	Elastic Modulus（GPa）	Poisson's ratio
undamaged layer	3.438	0.3
Damaged layer	3.13	0.3

FTIR spectra of PI film before and after UV irradiation was represented in Fig.S2. The intensity of the characteristic peak of C=O and C-N-C bands decreased remarkably after UV irradiation, which consisted with the increasing roughness of the PI film.

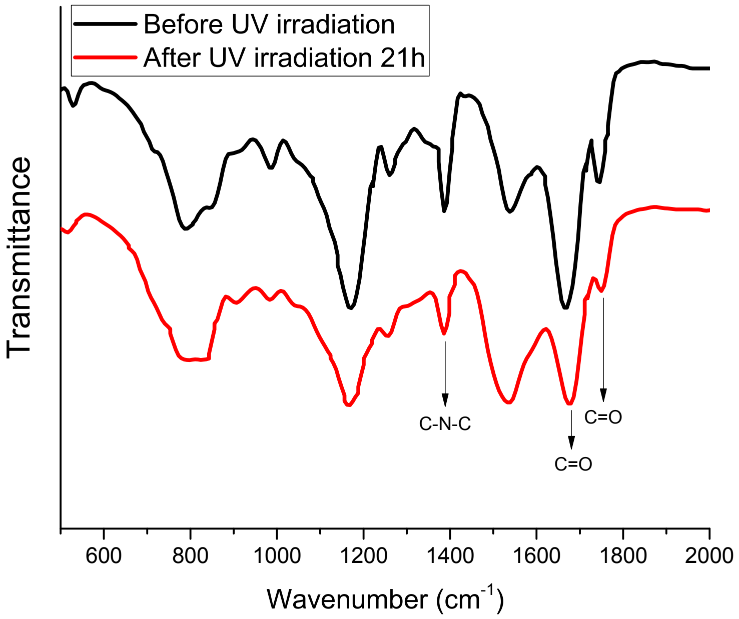

Fig.S2 FTIR spectra of PI film before and after UV irradiation
